# Effect of Response Surface Methodology-Optimized Ultrasound-Assisted Pretreatment Extraction on the Composition of Essential Oil Released From *Tribute citrus* Peels

**DOI:** 10.3389/fnut.2022.840780

**Published:** 2022-04-28

**Authors:** Guoqiang Li, Shuxun Liu, Qingqing Zhou, Jiarun Han, Cheng Qian, Yongquan Li, Xia Meng, Xin Gao, Tao Zhou, Ping Li, Qing Gu

**Affiliations:** Key Laboratory for Food Microbial Technology of Zhejiang Province, College of Food Science and Biotechnology, Zhejiang Gongshang University, Hangzhou, China

**Keywords:** *Tribute citrus*, essential oil, response surface methodology, hydrodistillation, ultrasound-assisted extraction, electronic nose

## Abstract

The traditional hydrodistillation (HD) and ultrasound-assisted pretreatment extraction (UAPE) methods were proposed to obtain essential oil (EO) from *Tribute citrus* (TC) peels. The Box-Behnken design was employed to optimize the HD and UAPE procedures. Moreover, gas chromatography-mass spectrometry (GC-MS) and electronic nose (E-nose) were applied to identify the discrepancy of the extraction methods. The yield of EO extracted by UAPE (114.02 mg/g) was significantly higher than that by HD (85.67 mg/g) (*p* < 0.01) undergoing 40 min short time-consuming UPAE. A total of 28 compounds were extracted from the TC peels as terpenes were the predominant components. d-Limonene was the most vital compound in the *T. citrus* essential oil (TCEO), accounting for 86.38% of the total volatile concentration in HD and 86.75% in UAPE, respectively, followed by α-pinene, sabinene, γ-myrcene, and β-phellandrene. The chart of radar and graphic of the principal component analysis by E-nose displayed no significance, which was similar to the GC-MS results. This study demonstrated that UAPE is an efficient and short time-consuming method for TCEO extraction, which provides a promising method for the separation of EO from aromatic plant materials.

## Introduction

**Figure 1:**
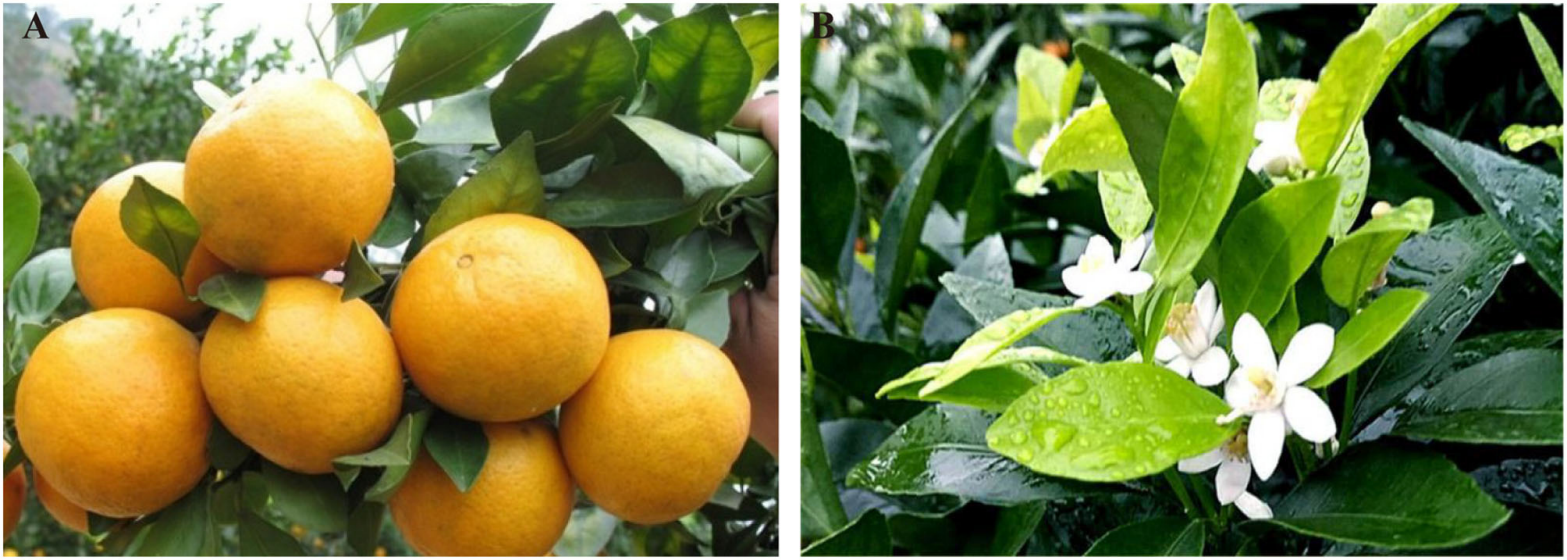
The graph of the TC fruits and flowers. **(A)** The fruits of TC; **(B)** the flowers of TC.

*Tribute citrus* (TC) is a hybrid variety of orange and tangerine and belongs to the genus *Citrus* of the Rutaceae family. TC is mainly distributed in the south of China; its fruits and flowers are shown in Figure 1. TC berry is characterized by its pleasant flavor and attractive appearance. Moreover, the abundance of carbohydrate, fiber, protein, and calcium in TC fresh has promoted the cultivation of TC for daily needs and disease remedy in the form of a traditional herb to a certain degree (1). The output of TC was 0.32 million tons in 2020, most of the TC had generally been eaten fresh, and approximately a third of TC was processed into drinks or distilled into liquor. The agro-food industry generated 30–50% of waste or by-products (2). Recently, much attention has been focused on the utilization of TC peel, which exhibits strong various functional properties, including antioxidant, antimicrobial, anticancer, anti-inflammatory, antiobesity, and antihyperglycemic activities (3, 4). While the left peel waste is a rich source of organic carbon, it has attracted much attention, but the problems of sustainability, bioavailability, and secondary pollution remain unsolved (5). More research regarding TC peel by-products has attracted public attention as well as environmental concerns.

Plant essential oil (EO), also known as volatile oil, is a secondary metabolite containing mixtures of lipophilic and volatile compounds with an aromatic odor extracted from different parts of the plants and also a wide diversity of chemical compounds, mainly terpenes (6–8). But its biological activities, percentage of food production, as well as its hydrophobic properties are not clear, which prevents its large-scale application (9). Plant EO is recognized for its effective, well-tolerated, and nontoxic characteristics, which have led to the development of natural compounds and their derivatives to ameliorate drug resistance and use in many industries, such as the food industry and the pharmaceutical industry, and as traditional domestic natural therapies for thousands of years (10, 11). Recently, an enormous amount of attention has been directed toward the use of EOs as antimicrobials and antioxidants in the food security matrix for the shelf life extension of food products due to the increased preference for natural products and the probability of distrust of synthetic chemicals (12, 13).

The traditional plant EO was universally extracted by hydrodistillation (HD) technique using Clevenger apparatus (14, 15). However, this conventional technique is extremely time- and energy-consuming; meanwhile, it facilitates low capacity and high grade of thermo possibly damaged by such harsh conditions. Therefore, the urgent development of new techniques for imminent extraction is of utmost importance (16, 17). The ultrasound-assisted pretreatment extraction (UAPE) as a pretreatment to HD of ground plant material that is proposed as a substitutable, green, environmentally-friendly, and efficient technique for the separation of plant EO without the addition of any organic solvents (18, 19). The promotion of plant EO separation using UAPE is mainly attributed to the generation and explosion of bubbles resulting from water vibration (20). UAPE gives rise to instantaneous increases in temperature and energy, promoting the inside of plant cells and thus disrupts these cells *via* the evaporation of EO through azeotropic distillation. Compared to the conventional HD, UAPE is a simple and cost-effective process that enhances the yield and quality of EO with a significant increase as well as reduces the consumption of time and energy (21, 22). The Clevenger apparatus steam distillation is the primary traditional HD for obtaining EO (23). However, highly novel and efficiently imminent diffusion technologies have emerged to obtain high-quality EO and address the drawbacks of the conventional mainstream process, which is of utmost importance. Therefore, developing an alternative, simple, and efficient extractable EO from citrus by UAPE would offer great ecological and economic benefits.

In this study, UAPE is proposed for TC peel EO extraction, and response surface methodology (RSM) was employed to determine and optimize the extraction parameters influencing the extraction efficiency through the Box-Behnken design (BBD). The verified superiority of the proposed method is further validated by comparison with the HD methods. Furthermore, gas chromatography-mass spectrometry (GC-MS) was employed to identify the constitutions of the TCEO isolated by the proposed UAPE and HD methods, and electronic nose (E-nose) was applied for discrepancy of discrimination. This study provides a foundation for further research on the functional properties of EO.

## Materials and Methods

### Materials and Chemicals

The TC berries were harvested in October 2020 in Wuming, China. The TC peels were detached manually and dried in an oven at 37°C for 72 h, and the moisture content of the TC peels was detected with a water activity meter at 4.12 ± 0.16% (METTLER TOLEDO, MJ33, China). The dried peels were placed in a dark at room temperature and then pulverized by the mill through a 60 mesh sieve and stored under a dark desiccator provisional before use. Ultrapure water was prepared using a Milli-Q water purification system (Millipore, Waltham, MA, USA) that was used in the whole study. The TC peel suspension was disrupted by sonication using a Sonics Vibra Cell sonicator (JY92-IIN, Ningbo Scientz Biotechnology Co., Ltd., Ningbo, China) working for 5 s with 10 s intervals and 400 W operation power at 20 kHz for a total of 20 min, and in case of high temperature, ice was placed around the flask aiming to mitigate the destruction of chemical components in TC due to high temperature, the ice was placed around the flask. All chemicals and reagents used in this experiment were of the analytical grades and were purchased from Shanghai Maikelin Biotechnology Co., Ltd, Shanghai, China.

### EO Extraction

#### Conventional HD Extraction

TCEO extraction with a sodium chloride solvent was applied with slight modifications according to the method described by Krishna P. Solanki (24). Ten grams of fine homogenized TC powder was mixed with a 1.5% sodium chloride aqueous solution. The RSM based on the extraction conditions (concentration of sodium chloride, extraction time, solvent-solid rate) was defined by a single-factor test and BBD. The whole distillation process was accomplished until there was no more significant increase in the volume of the EO. The yield was determined per gram of the raw material (dry weight). Three replicates were carried out for each treatment. The TCEO was stored at −18°C until analysis.

#### Ultrasound-Assisted Extraction

The mesh sieved TC homogeneous fine powder was mixed with a 1.5% sodium chloride solution, then operated in an ultrasound extraction equipment, working at a frequency of 455 kHz, and the established solvent-solid ratio, ultrasound time, extraction time, and concentration of sodium chloride. Then, like the subsequent steps of traditional HD extraction in this study, the TCEO was obtained and stored at −18°C for further analysis.

### TCEO Yield Determination

The yield of TCEO was calculated as


(1) $$\begin{array}{r} \text{Essential oil yield},\text{ mg}/\text{g}=\frac{\text{Essential oil mass}}{\text{TC powder mass}} \end{array}$$

### Experimental Design

#### Single-Factor Test

The impacts of different sodium chloride concentrations, solvent-solid ratios, extraction times, and ultrasound times on the EO yield were determined. In this study, the extraction conditions performed were the concentration of sodium chloride (0.0, 0.5, 1.0, 1.5, 2.0, 2.5, 3.0%), solvent-solid ratio (10:1, 12:1, 14:1, 16:1, 18:1, 20:1, 22:1 24:1), extraction time (30, 60, 90, 120, 150, 180 min), and ultrasound time (5, 10, 15, 20, 25, 30 min).

#### Response Surface Design

For the traditional HD extraction method, the single-factor test was performed to investigate the effects of concentration of sodium chloride (X_1_), extraction time (X_2_), and solvent-solid rate (X_3_) on the extraction efficiency. The optimal extraction process was determined with BBD. For UAPE, the effects of the solvent-solid ratio (X_4_), ultrasound time (X_5_), extraction time (X_6_), and concentration of sodium chloride (X_7_) on the TCEO extraction process were investigated. Y presents the response value of the design TCEO yield (mg/g). Each variable was coded at three levels (low, central, high), which are referred to as −1, 0, and 1, respectively. The actual experiment runs implemented in this study were operated using the Design Expert software, consisting of 5 central points and 12 middle points that were circumscribed on the edges of a cube, and the corresponding results are given in Tables 1, **3**. TCEO extraction was digitally represented by the following second-order polynomial equation [Equation (2)], and the yield of EO was shaped by the multiregression process.


(2) $$\begin{array}{r} Y=\beta_{0}+\overset{n}{\underset{i=1}{\sum }}\beta_{\text{i}}X_{\text{i}}+\overset{n}{\underset{i=1}{\sum }}\beta_{\text{ii}}X_{\text{i}}^{2}+\overset{n}{\underset{i=1}{\sum }}\beta_{\text{ij}}X_{\text{i}}X_{\text{j}} \end{array}$$

where Y is the EO yield of three tests for each treatment; β_0_, β_i_, β_ii_, and β_ij_ represent the corresponding regression coefficients of the intercept, linear, quadratic, and interactive terms, respectively; and X_i_ and X_j_ are the coded independent variables.

**Table 1:** Box-Behnken experimental design matrix with experimental response for TCEO using traditional hydrodistillation extraction.

**Runs**	**Solvent-solid ratio (X_**1**_, -)**	**Sodium Chloride concentration (X_**2**_, %)**	**Extraction time (X_**3**_, min)**	**Essential oil yield (Y_**1**_, mg/g)**
1	22 (+1)	1.5 (0)	90 (−1)	75.53 ± 0.74
2	20 (0)	1.5 (0)	120 (0)	84.97 ± 0.87
3	20 (0)	1 (−1)	150 (+1)	83.30 ± 1.70
4	20 (0)	2 (+1)	150 (+1)	71.53 ± 1.94
5	20 (0)	1 (−1)	90 (−1)	76.67 ± 1.51
6	20 (0)	1.5 (0)	120 (0)	86.40 ± 0.73
7	20 (0)	1.5 (0)	120 (0)	85.67 ± 2.48
8	20 (0)	1.5 (0)	120 (0)	84.37 ± 1.48
9	18 (-1)	2 (+1)	120 (0)	73.77 ± 0.19
10	18 (-1)	1.5 (0)	150 (+1)	72.93 ± 2.66
11	22 (+1)	1.5 (0)	150 (+1)	65.10 ± 1.71
12	18 (-1)	1.5 (0)	90 (−1)	62.87 ± 3.07
13	22 (+1)	1 (−1)	120 (0)	78.90 ± 0.70
14	18 (-1)	1 (−1)	120 (0)	67.00 ± 1.15
15	20 (0)	2 (+1)	90 (−1)	82.03 ± 1.72
16	22 (+1)	2 (+1)	120 (0)	66.87 ± 1.47
17	20 (0)	1.5 (0)	120 (0)	84.27 ± 2.78

### Gas Chromatography-Mass Spectrometry Analysis

The chemical constituents of the TCEO extracted by both UAPE and HD were measured on a 7890A-5975C-mass chromatograph (Agilent, USA) fitted with a split injector. A DB-WAX MS column [60 m × 0.25 mm (i.d.), the film thickness of 0.25 μm] was employed for the separation of the volatile compounds. The programmed column temperature was the initial temperature of 35°C for 5 min, increasing at a constant ramp at 5°C/min to 240°C, then held for 5 min and stopped at an ultimate temperature of 230°C. The gas type was helium, and the flow rate was maintained at a constant of 1 ml/min. The determination process was operated in EI mode with a 70 eV electron energy voltage. The detector was preset at a scan range of 33–500 amu. The injection and interface temperatures were 240 and 80°C, respectively. GC-MS analysis was programmed to perform for a 1.0 μl sample in split mode (split ratio, 1:200). Most components of the EOs extracted by each method were identified on the basis of retention index (Kovat′s RI) and the NIST14 Mass Spectral Library.

### E-Nose Analysis

The E-nose generally consists of a gas sensor array utilized to detect and distinguish odors of samples through multiple sensors. The discrimination of TCEO extracted by HD and UAPE was measured with an electronic nose. Spiked 500 μl of TCEO was put into a 10 ml flask at 60 °C water bath, which was then removed after 40 min heating and equilibrium for 10 min at room temperature. E-nose conditions were employed as an initial sample flow rate 600 μl/min, sampling interval 1 s, washing time 120 s, detection time 200 s, and trimming zero time 1 s, and each sample was repeated in sextuplicate.

### Statistical Analysis

Design Expert 8.0 software (Stat-Ease, Minneapolis, USA) was employed to perform BBD. ANOVA was applied to determine the statistically significant differences between the compared data in the TCEO yield.

All experiments were performed in triplicate. A BBD matrix comprising 29 trials was formulated with Design Expert software. The real responses of each experiment matrix run through BBD are expressed as the average values that were grounded on the built-in default settings in Design Expert 8.0 software, and the others are represented as the mean values ± SD. The level of confidence required for significance was set at *p* < 0.05. The identified TCEO was analyzed by GC-MS, and the analysis discrimination of TCEO was employed with an E-nose.

## Results

### Single-Factor Test: The Effect of Traditional HD Extraction on the TCEO Yield

**Figure 2:**
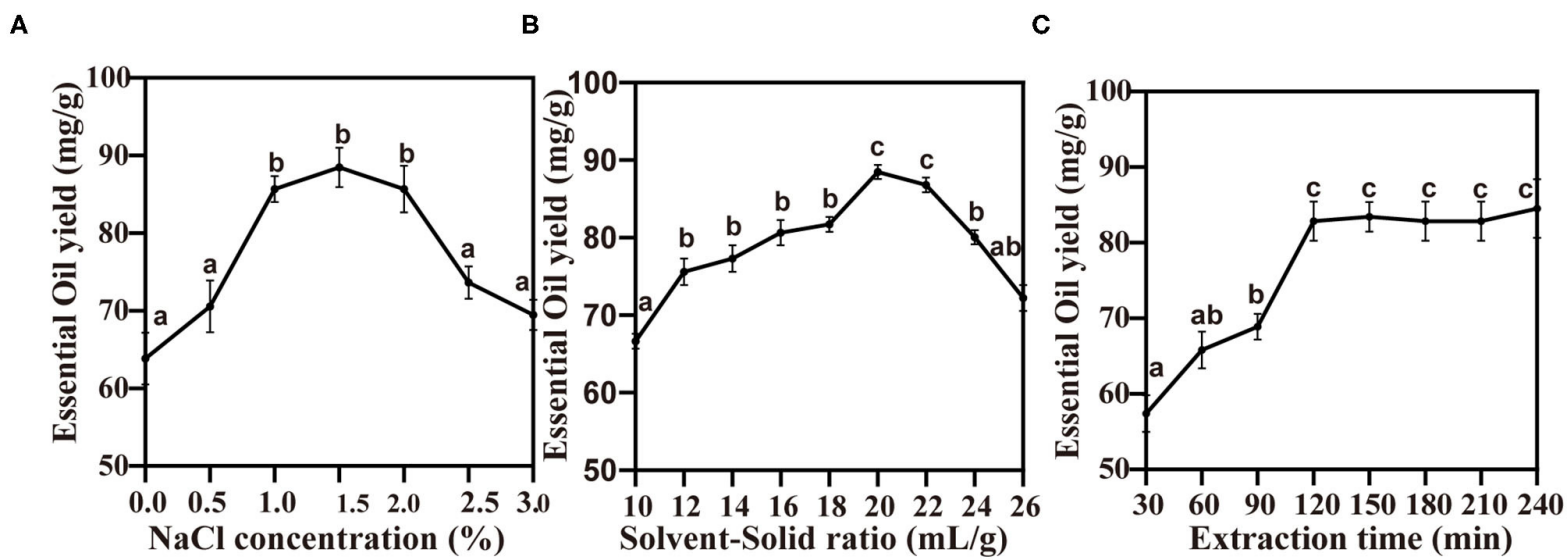
The effect of the traditional hydrodistillation extraction on the TCEO yield. **(A)** The concentration of sodium chloride on the TCEO yield; **(B)** the solvent-solid ratio on the TCEO yield; **(C)** the extraction time on the TCEO yield. Values with the same letter are not significantly different *p* < 0.05 according to ANOVA.

The EO extraction is largely linked to the components and materials (24). According to previous studies, plant cell walls were the most resistant to release Eos (25). Therefore, sodium chloride was used to change the cytomembrane permeability (26). The results show that the maximum TCEO yield was obtained at a concentration of sodium chloride 1.5%, followed by the EO yield falling dramatically as the concentration of sodium chloride rose (Figure 2A). Therefore, 1.5% sodium chloride was selected as the optimum concentration for the extraction of the TCEO. The result of the solvent-solid ratio on the yield of TCEO is shown in Figure 2B. The EO yield increased significantly with an increase in solvent-solid ratio of 20:1. Hereby, the 20:1 ratio was selected as the best solvent-solid ratio. Figure 2C shows the effect of extraction time on the yield of TCEO. The TCEO yield increased significantly during extraction time from 60 to 120 min, while, thereafter, no significant difference in the yield of EO was shown. The result indicates that the TCEO has been extracted to the greatest extent at 150 min.

### Optimization of the Traditional HD Extraction Process

In consideration of the results of the single-factor test, the effects of sodium chloride concentration (X_1_), extraction time (X_2_), and solvent-solid ratio (X_3_) on the TCEO yield were investigated using the BBD (Table 1). A multiple regression fitting to obtain a quadratic polynomial equation that depicts the relationship between each response value and test variables is as follows:


(3) $$\begin{array}{l} Y_{1}=85.14+1.23*X_{1}-1.46*X_{2}-0.53*X_{3}-4.70*X_{1}*X_{2}\\ \;-5.12*X_{1}*X_{3}-4.28*X_{2}*X_{3}-11.39*X_{1}^{2}-2.11*X_{2}^{2}\\ \;-4.64*X_{3}^{2} \end{array}$$

where Y_1_ represents the TCEO yield, and X_1_, X_2_, and X_3_ are the actual values of the independent variables. The effects of extraction parameters on the TCEO yield are displayed on the ANOVA table (Table 2); the regression model is significant (*p* < 0.001), lack of fit is not significant, R^2^ = 99.51%, adj-R^2^ = 98.87%, and the difference between the R^2^ and the adj-R^2^ values observed for all variables indicates that the proposed models are reasonable to fit the experimental data. The *t*-test and p-values indicate that the independent variable solvent-solid ratio and sodium chloride concentration have a significant effect on the TCEO yield (*p* < 0.05). The interactive terms with higher F-values and lower p-values exhibit a bulky significance and are capable of verifying a reasonable fitting model. The adequacy precision value of 33.887 indicates that the proposed model could be applied to navigate the design region.

**Table 2:** The ANOVA of the fitting model of TCEO by traditional hydrodistillation extraction.

**Source**	**Sum of squares**	**DF**	**Mean square**	**F value**	***p*-value**	**Significance**
Model	9.99	9	1.11	157.93	<1E-4	^**^
X_1_-solvent-solid ratio	0.12	1	0.12	17.08	4.4E-3	^**^
X_2_-sodium chloride concentration	0.17	1	0.17	24.35	1.7E-3	^**^
X_3_-extraction time	0.023	1	0.023	3.29	0.1126	ns
X_1_X_2_	0.88	1	0.88	125.73	<1E-4	^**^
X_1_X_3_	1.04	1	1.04	148.04	<1E-4	^**^
X_2_X_3_	0.73	1	0.73	104.02	<1E-4	^**^
X${}_{1}^{2}$	5.46	1	5.46	777.59	<1E-4	^**^
X${}_{2}^{2}$	0.19	1	0.19	26.86	1.3E-3	^**^
X${}_{3}^{2}$	0.92	1	0.92	130.52	<1E-4	^**^
Residual	0.049	7	7.028E-003			
Lack of Fit	0.017	3	5.825E-003	0.73	0.5835	ns
Pure Error	0.032	4	7.930E-003			
Cor Total	10.04	16				
Std. Dev. Mean	C.V. %	PRESS	R^2^	Adj R^2^	Pred R^2^	Adeq precision
0.84 76.60	1.10	32.28	0.9951	0.9887	0.9678	33.887

** *p < 0.01, very significant*;

* p < 0.05, significant.

**Figure 3:**
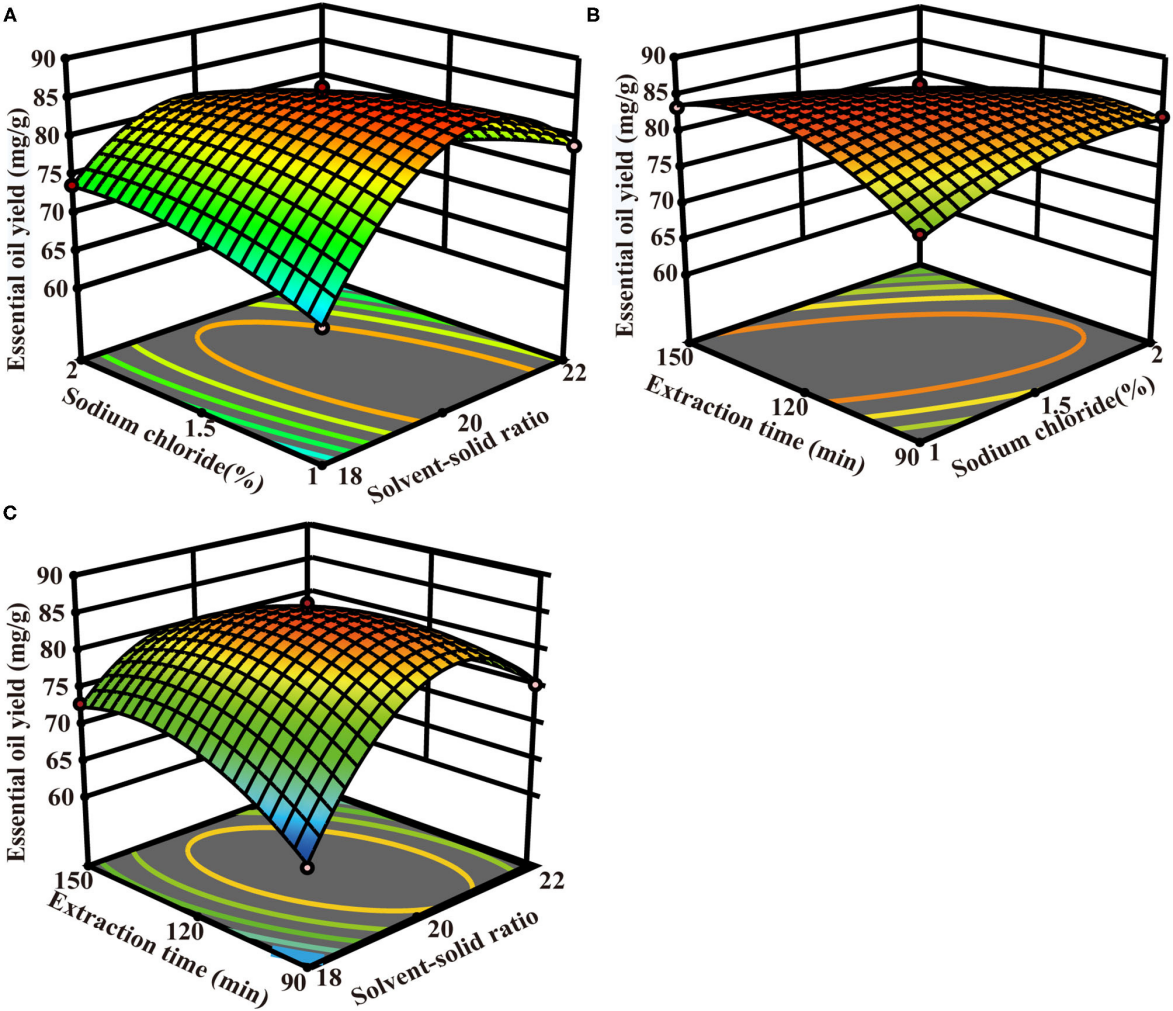
The optimization of the HD extraction process. **(A)** The interaction of solvent-solid ratio and sodium chloride concentration; **(B)** the interaction of sodium chloride concentration and extraction time; **(C)** the interaction of solvent-solid ratio and extraction time.

Also, it was observed that the interactive effects of the solvent-solid ratio and sodium chloride concentration (X_1_:X_2_), solvent-solid ratio and extraction time (X_1_:X_3_), sodium chloride concentration, and extraction time (X_2_:X_3_) were significant (*p* < 0.01). All the three-square terms (X${}_{1}^{2}$, X${}_{2}^{2}$, and X${}_{3}^{2}$) have tremendous significant impacts on the TCEO yield (*p* < 0.01) and show a parabolic trend in TCEO extraction. Herein, the extraction time (X_3_) did not show any statistical significance. Furthermore, a series of three-dimensional (3D) surface plots were generated where the third independent variable was kept constant and varying the other two to determine the interactive effects of the independent variables on the response (27). As shown in Figure 3A, the effect of the solvent-solid ratio showed a quadratic effect on the TCEO yield in a lower solvent-solid ratio range while decreasing with the increasing solvent-solid ratio in a higher range, and the sodium chloride showed a slight quadratic effect on the TCEO yield. A quadratic effect of extraction time and sodium chloride concentration on the TCEO yield was observed as shown in Figure 3B. As depicted in Figure 3C, the solvent-solid ratio exerted a quadratic effect on the TCEO yield in a lower solvent-solid ratio range while decreasing with the increasing solvent-solid ratio in a higher range, and the extraction time showed a slight quadratic effect on the TCEO yield. In summary, the optimal conditions for the traditional HD extraction process were 20.32:1 solvent-solid ratio, 125.38 min extraction time, and sodium chloride concentration 1.15%, yielding a TCEO of 85.69 mg/g. Based on the predicted conditions, the verification experiment was carried out with a 20.32:1 solvent-solid ratio at the sodium chloride concentration 1.15% and 125.38 min extraction time. The TCEO yield was 85.67 mg/g, which was similar to the predicted value, hence conforming to the model validity.

### Single-Factor Test: The Effect of UAPE on the TCEO Yield

**Figure 4:**
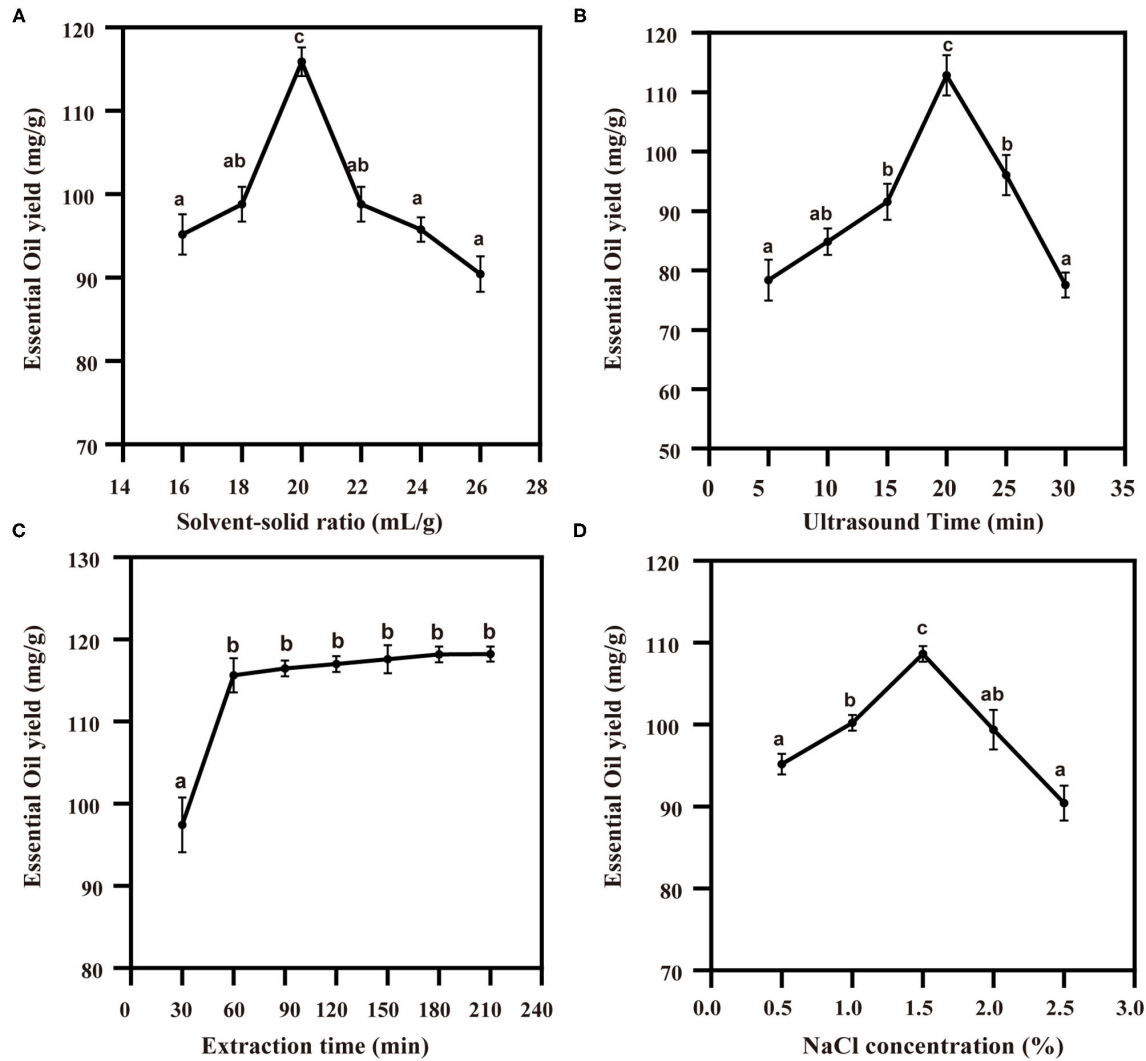
The effect of the ultrasound-assisted extraction on the TCEO yield. **(A)** The concentration of solvent-solid ratio on the TCEO yield; **(B)** ultrasound time on the TCEO yield; **(C)** the extraction time on the TCEO yield; **(D)** the sodium chloride on the TCEO yield. Values with the same letter are not significantly different *p* < 0.05 according to ANOVA.

The result of the solvent-solid ratio on the yield of TCEO is shown in Figure 4A. With the increase in solvent-solid ratio, the EO yield increased significantly at the ratio of 20:1. To avoid solution waste and facilitate subsequent operation, 20:1 was selected as the best solvent-solid ratio. The effect of ultrasound time on the yield of TCEO is displayed in Figure 4B; the maximum TCEO yield was obtained at 20 min, followed by the EO yield decrease as ultrasound time extended. The effect of extraction time on the yield of TCEO is shown in Figure 4C. With the increase in extraction time, the TCEO yield increased significantly, and after 60 min, the yield of EO did not change significantly, which indicates that the TCEO has been extracted to the greatest extent. The results show that the maximum TCEO yield was obtained at the concentration of sodium chloride 1.5%, followed by the EO yield falling dramatically as the concentration of sodium chloride rose (Figure 4D). Therefore, 1.5% sodium chloride was selected as the optimum concentration for the extraction of the TCEO.

### Process Optimization of UAPE

Ultrasound was used as an assisted method to explore the conditions of TCEO extraction, and the effects of the solvent-solid ratio (X_4_), ultrasound time (X_5_), extraction time (X_6_), and sodium chloride concentration (X_7_) on the TCEO yield were observed using a BBD. The results obtained after the 27 runs, along with the complete experimental design, are shown in Table 3. A multiple regression equation was obtained that represents an empirical relationship between the TCEO yield and the process variables as given:


(4) $$\begin{array}{l} Y_{2}=114.02+2.98*X_{4}+3.33*X_{5}+1.50*X_{6}+6.10*X_{7}\\ \;-9.33c*X_{4}*X_{5}+0.75*X_{4}*X_{6}-2.57*X_{4}*X_{7}\\ \;-5.80*X_{5}*X_{6}+3.17*X_{5}*X_{7}-2.75*X_{6}*X_{7}\\ \;-11.56*X_{4}^{2}-13.16*X_{5}^{2}-13.18*X_{6}^{2}-13.06*X_{7}^{2} \end{array}$$

where Y_2_ represents the TCEO yield, and X_4_, X_5_, X_6_, and X_7_ are the actual values of the independent variables. The effects of extraction parameters on the TCEO yield are displayed on the ANOVA table, as can be seen from Table 4; the regression model is significant (*p* < 0.001), lack of fit is not significant, R^2^ = 95.44%, adj-R^2^ = 90.87%, and the difference between the R^2^ and the adj-R^2^ values is observed for all variables, indicating that the proposed models are reasonable to fit the experimental data. The *t*-test and *p*-values indicate that the independent variable solvent-solid ratio, ultrasound time, and sodium chloride concentration have a significant effect on the TCEO yield (*p* < 0.05). The interactive terms with higher F-values and lower *p*-values exhibit a bulky significance and are capable of verifying a reasonable fitting model. The adequacy precision value of 15.187 indicates that the proposed model could be applied to navigate the design region.

**Table 3:** Box-Behnken experimental design matrix with experimental response for TCEO using ultrasound-assisted extraction.

**Runs**	**Solvent-solid ratio (X_**4**_, -)**	**Ultrasound time (X_**5**_, %)**	**Extraction time (X_**6**_, min)**	**Concentration sodium chloride (X_**7**_, %)**	**Essential oil yield (Y_**2**_, mg/g)**
1	22 (+1)	20 (0)	60 (0)	1 (−1)	90.9 ± 1.8
2	20 (0)	25 (+1)	90 (+1)	1.5 (0)	86.9 ± 1.4
3	20 (0)	20 (0)	30 (0)	1 (−1)	76.7 ± 3.2
4	22 (+1)	20 (0)	90 (+1)	1.5 (0)	92.7 ± 1.8
5	22 (+1)	20 (0)	60 (0)	2 (+1)	102.0 ± 3.8
6	22 (+1)	15 (−1)	60 (0)	1.5 (0)	95.1 ± 2.1
7	20 (0)	20 (0)	30 (-1)	2 (+1)	95.7 ± 2.5
8	20 (0)	25 (+1)	30 (-1)	1.5 (0)	97.0 ± 1.9
9	18 (−1)	25 (+1)	60 (0)	1.5 (0)	103.3 ± 4.1
10	20 (0)	15 (0)	90 (+1)	1.5 (0)	92.0 ± 2.4
11	22 (+1)	25 (+1)	60 (0)	1.5 (0)	85.5 ± 2.1
12	20 (0)	20 (0)	60 (0)	1.5 (0)	113.5 ± 2.3
13	20 (0)	20 (0)	60 (0)	1.5 (0)	118.6 ± 2.0
14	20 (0)	20 (0)	60 (0)	1.5 (0)	112.3 ± 2.9
15	18 (−1)	20 (0)	30 (-1)	1.5 (0)	84.3 ± 2.5
16	20 (0)	15 (−1)	60 (0)	2 (+1)	84.4 ± 1.6
17	20 (0)	20 (0)	90 (+1)	2 (+1)	94.4 ± 1.8
18	20 (0)	25 (+1)	60 (0)	2 (+1)	95.1 ± 0.4
19	18 (−1)	20 (0)	90 (+1)	1.5 (0)	86.1 ± 3.8
20	18 (−1)	20 (0)	60 (0)	1 (−1)	73.8 ± 2.0
21	20 (0)	20 (0)	90 (+1)	1 (−1)	86.4 ± 1.8
22	20 (0)	15 (-1)	30 (-1)	1.5 (0)	78.9 ± 3.0
23	20 (0)	20 (0)	60 (0)	1.5 (0)	110.4 ± 2.1
24	22 (+1)	20 (0)	30 (-1)	1.5 (0)	87.9 ± 1.4
25	20 (0)	25 (+1)	60 (0)	1 (−1)	81.8 ± 5.7
26	20 (0)	20 (0)	60 (0)	1.5 (0)	115.3 ± 2.2
27	18 (−1)	20 (0)	60 (0)	2 (+1)	95.1 ± 0.4
28	18 (−1)	15 (−1)	60 (0)	1.5 (0)	75.5 ± 1.3
29	20 (0)	15 (−1)	60 (0)	1 (−1)	83.8 ± 1.0

**Table 4:** The ANOVA of the fitting model of TCEO by ultrasound-assisted extraction.

**Source**	**Sum of squares**	**df**	**Mean square**	**F value**	***p*-value**	**Significance**
Model	3,995.579	14	285.3984721	20.90944361	<1E-4	^**^
X_4_-solvent-solid ratio	106.8033	1	106.8033333	7.824843136	0.0143	^*^
X_5_-ultrasound time	133.3333	1	133.3333333	9.768537981	7.4E-3	^**^
X_6_-extraction time	27	1	27	1.978128941	0.1814	ns
X_7_-sodium chloride concentration	446.52	1	446.52	32.71385685	<1E-4	^**^
X_4_X_5_	347.8225	1	347.8225	25.48287976	2E-4	^**^
X_4_X_6_	2.25	1	2.25	0.164844078	0.6909	ns
X_4_X_7_	26.5225	1	26.5225	1.943145365	0.1851	ns
X_5_X_6_	134.56	1	134.56	9.858408531	7.2E-3	^**^
X_5_X_7_	40.3225	1	40.3225	2.954189046	0.1077	ns
X_6_X_7_	30.25	1	30.25	2.216237055	0.1587	ns
X${}_{4}^{2}$	866.1878	1	866.1877883	63.46041232	<1E-4	^**^
X${}_{5}^{2}$	1,122.655	1	1,122.654815	82.25022152	<1E-4	^**^
X${}_{6}^{2}$	1,126.926	1	1,126.925626	82.56311836	<1E-4	^**^
X${}_{7}^{2}$	1,105.653	1	1,105.652653	81.00457452	<1E-4	^**^
Residual	191.0897	14	13.6492619			
Lack of Fit	152.1417	10	15.21416667	1.562510698	0.3540	ns
Pure Error	38.948	4	9.737			
Cor Total	4,186.668	28				
Std. Dev. Mean	C.V. %	PRESS	R^2^	Adj R^2^	Pred R^2^	Adeq Precision
3.69 92.94	3.98	937.19	0.9544	0.9087	0.7761	15.187

** *p <0.01, very significant*;

* *p <0.05, significant*.

**Figure 5:**
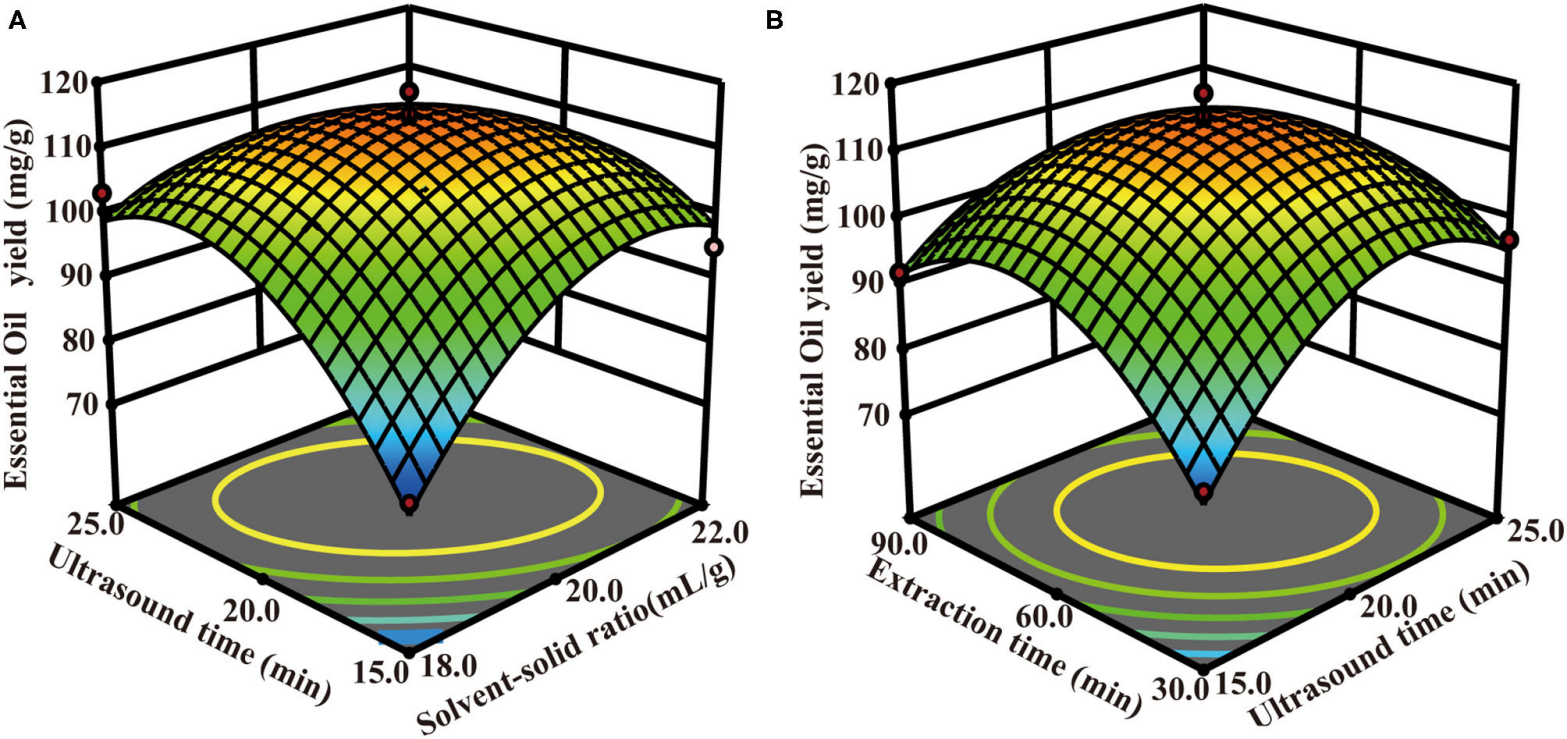
The optimization of the UAPE extraction process. **(A)** The interaction of solvent-solid ratio and ultrasound time; **(B)** the interaction of ultrasound time and extraction time.

Also, it was observed that the interactive effects of the solvent-solid ratio and ultrasound time (X_4_:X_5_), ultrasound time, and extraction time (X_5_:X_6_) were significant (*p* < 0.01). All the four-square terms (X${}_{4}^{2}$, X${}_{5}^{2}$, X${}_{6}^{2}$, and X${}_{7}^{2}$) have tremendous significant impacts on the TCEO yield (*p* < 0.01) and show a parabolic trend in TCEO extraction. Herein, the extraction time (X_6_) did not show any statistical significance. Furthermore, a series of 3D surface plots were generated where the third independent variable was kept constant and varying the other two to determine the interactive effects of the independent variables on the response. As shown in Figure 5A, the effect of the solvent-solid ratio and ultrasound time shows a quadratic effect on the TCEO yield in a lower solvent-solid ratio range while decreasing with the increasing solvent-solid ratio and ultrasound time in a higher range. As depicted in Figure 5B, the ultrasound time and extraction time exerted a quadratic effect on the TCEO yield in a lower solvent-solid ratio range while decreasing with the increasing solvent-solid ratio in a higher range. In summary, the optimal conditions for the ultrasound-assisted extraction process were 20.09:1 solvent-solid ratio, 20.7 min ultrasound-assisted, extraction 60.04 min, and sodium chloride concentration 1.62%, yielding a TCEO of 115.072 mg/g. Based on the predicted conditions, the verification experiment was carried out with a 20:1 solvent-solid ratio at the sodium chloride concentration 1.62%, ultrasound-assisted 20.7 min, and extraction 60 min. The TCEO yield was 114.02 mg/g, which was similar to the predicted value, hence conforming to the model validity.

#### Investigation of Model Adequacy

**Figure 6:**
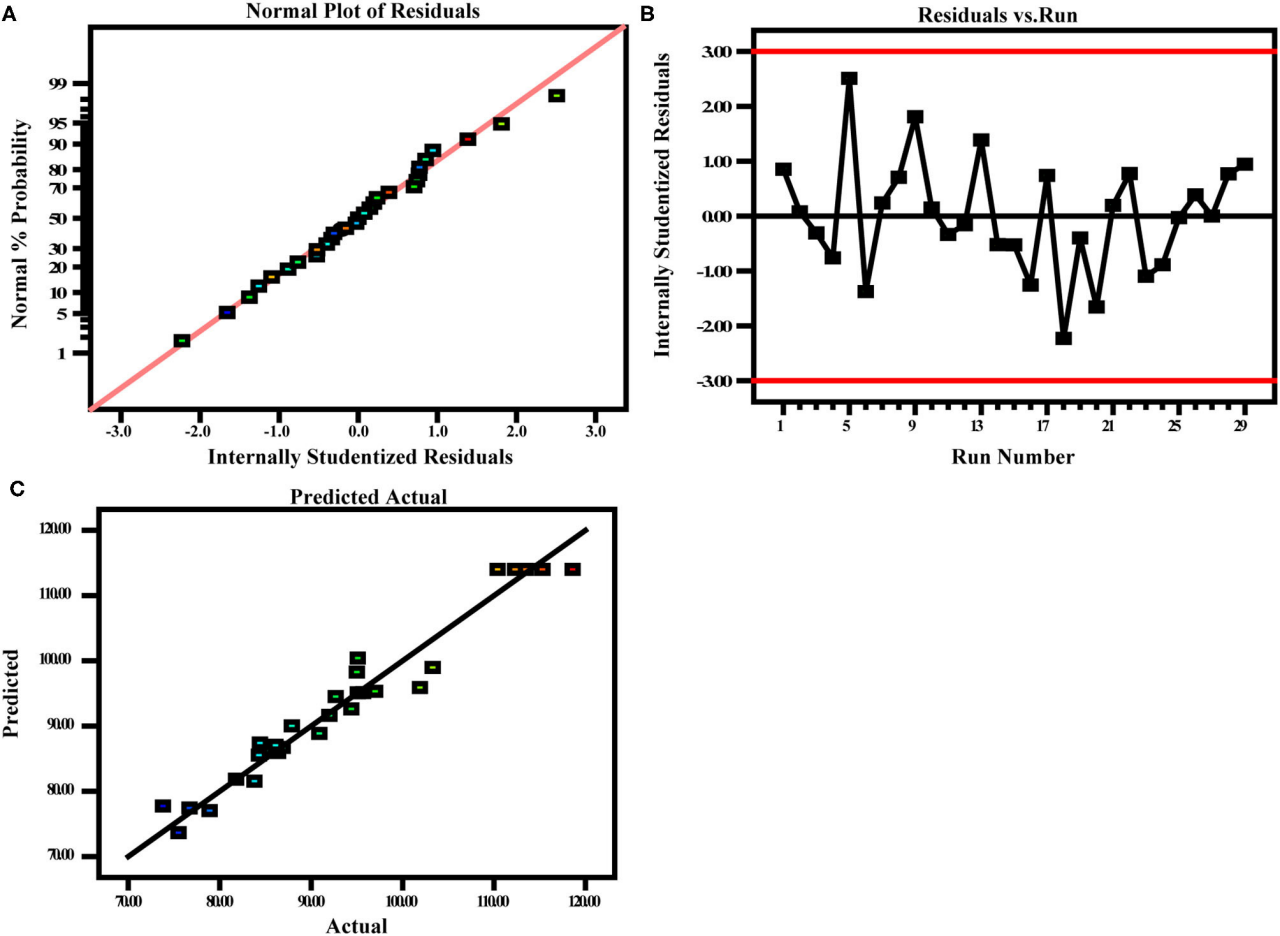
Three diagnostic plots for model adequacy checking. Normal plots **(A)**, residuals vs. run number **(B)**, and predicted vs. actual **(C)**.

Figure 6 depicts three residual plots of the ultrasound-assisted method, namely, the normal plot of residuals (A), residuals vs. run number (B), and predicted vs. actual (C). The normal plot of residuals (Figure 6A) displays an approximately straight line, indicating that the residuals follow a normal distribution and are independent of each other. The normal probability axis distributed density around the center and less at both ends, which indicates the data performance normally. Residuals vs. run number plot (Figure 6B) depicts a random scatter of data points (between +3.93041 and −3.93041) around the central line, indicating that the residuals conform to the normal distribution, which depicts that the quadratic model builds a connection between the causal factors and the TCEO yield. The predicted vs. actual plot (Figure 6C) shows a straight line surrounded by all data points, which indicates that the developed model was capable of accurately predicting the actual response values. In brief, the three residual plots show that the proposed model has a good adequacy to optimize the extraction of TCEO.

#### Method Validation

The desirability index is a very useful tool for obtaining the optimal process conditions that maximize the response on the count of the experimental data and model-predicted value (28). The optimal UAPE process conditions obtained from the BBD were the solvent-solid ratio of 20.09:1, sodium chloride concentration of 1.62%, ultrasound-assisted of 20.7 min, and extraction time of 60.04 min. Under these conditions, the maximum predicted TCEO yield was 115.072 mg/g, giving a desirability of 1, which represents full desirability. Under the above conditions, the mean TCEO yield of 114.02 ± 2.79 mg/g was obtained in good accordance with the actual experiments, which demonstrated that the acquired results are according to the predicted value. This result verifies the precision of the mathematical description of EO extraction by UAPE.

### Comparison of UAPE and HD

Compared with the extraction yield of traditional HD (85.67 ± 0.73 mg/g), higher EO yields were acquired by the proposed UAPE (114.02 ± 2.79 mg/g) in a shorter extraction time. The extraction time between the two techniques was 120 and 80 min for HD and UAPE, respectively. According to Table 5, a total of 28 constitutions were identified by GC-MS, and the types and percentages of each component of EO are displayed. A comparison of types was mainly terpenes, sesquiterpenes, aldehydes, alcohols, and long-chain terpenes. The percentages of TCEO extracted by the two methods for HD and UAPE were cyclic monoterpenes (90.99, 91.16%), acyclic monoterpenes (5.09, 4.94%), acyclic sesquiterpenes (0.45, 0.47%), aldehydes (1.17, 1.08%), alcohols (0.59, 0.56%), dicyclic monoterpenes (0.64, 0.62%), sesquiterpenes (0.07, 0.02%), cyclic terpene (0.5, 0.66%), and open-chain monoterpene (0.5, 0.53%), respectively. d-Limonene was the predominant constitution in TCEO, occupying 86.38 and 86.75%. The same results were found in a previous study; a variety of citrus were pretreated and grounded, then extracted by HD, supercritical CO_2_, molecular distillation, and organic solvent extraction (29–31). Besides limonene, the distribution of other chemical constituents in grapefruit, mandarin, lemon, and sweet orange was found, including the minor components (29).

**Table 5:** Chemical composition of TCEO by GC-MS analysis.

**No.^a^**	**compounds**	**RT (min)**	**CAS number**	**Molecular formula**	**Relative peak area (%)**		**RI^b^**	**Identification**
					**HD**	**UAPE**		
1	α-Pinene	10.13	2437-95-8	C_10_H_16_	1.77 ± 0.01	1.69 ± 0.07	1025	RI,MS
2	β-Pinene	12.5	127-91-3	C_10_H_16_	0.1 ± 0.01	0.10 ± 0.01	1114	RI,MS
3	Sabinene	12.8	3,387-41-5	C_10_H_16_	1.55 ± 0.24	1.47 ± 0.24	1125	RI,MS
4	α-Phellandrene	14.1	99-83-2	C_10_H_16_	0.15 ± 0.00	0.14 ± 0.00	1171	RI,MS
5	D-Limonene	15.4	5989-27-5	C_10_H_16_	86.38 ± 0.31	86.75 ± 0.23	1215	RI,MS
6	β-Phellandrene	15.54	555-10-2	C_10_H_16_	1.04 ± 0.00	1.01 ± 0.01	1221	RI,MS
I. Cyclic monoterpenes					90.99	91.16		
7	β-Myrcene	13.85	123-35-3	C_10_H_16_	5.09 ± 0.06	4.94 ± 0.04	1162	RI,MS
II. Acylic monoterpenes					5.09	4.94		
8	β-Famesene	27.17	18794-84-8	C_10_H_16_	0.04 ± 0.01	0.04 ± 0.00	1665	RI,MS
9	α-Farnesene	29.09	502-61-4	C_15_H_24_	0.41 ± 0.02	0.43 ± 0.02	1747	RI,MS
III. Acyclic sesquiterpenes					0.45	0.47		
10	Octanal	17.46	124-13-0	C_8_H_16_O	0.17 ± 0.02	0.16 ± 0.01	1289	RI,MS
11	Nonanal	20.36	124-19-6	C_9_H_18_O	0.05 ± 0.00	0.05 ± 0.00	1394	RI,MS
12	Decanal	23.15	112-31-2	C_10_H_20_O	0.20 ± 0.00	0.19 ± 0.01	1501	RI,MS
13	Dodecanal	28.3	112-54-9	C_10_H_16_	0.07 ± 0.00	0.07 ± 0.01	1713	RI,MS
14	Citronellal	22.61	2385-77-5	C_10_H_18_O	0.21 ± 0.01	0.2 ± 0.01	1480	RI,MS
15	Sinensal	40.81	17909-77-2	C_15_H_22_O	0.47 ± 0.11	0.41 ± 0.07	2183	RI^c^, MS
IV. Aldehydes					1.17	1.08		
16	Linalool	24.52	78-70-6	C_10_H_18_O	0.59 ± 0.07	0.56 ± 0.00	1556	RI,MS
V. Alcohols					0.59	0.56		
17	3-Carene	16.39	498-15-7	C_10_H_16_	0.2 ± 0.03	0.21 ± 0.03	1251	RI,MS
18	α-Muurolene	28.97	31983-22-9	C_15_H_24_	0.09 ± 0.01	ND^d^	1742	RI,MS
19	Bicyclogermacren	29.32	24703-35-3	C_15_H_24_	0.12 ± 0.02	0.14 ± 0.00	1757	RI,MS
20	d-Cadinene	29.68	483-76-1	C_15_H_24_	0.23 ± 0.02	0.27 ± 0.00	1773	RI,MS
VI. Dicyclic monoterpene					0.64	0.62		
21	Humulene	27.88	6753-98-6	C_10_H_16_	0.05 ± 0.03	ND^d^	1694	RI,MS
22	sesquiphellandrene	29.85	20307-83-9	C_15_H_24_	0.02 ± 0.00	0.02 ± 0.00	1781	RI,MS
VII. Sesquiterpene					0.07	0.02		
23	limoneneoxide	22.34	4959-35-7	C_10_H_16_O	0.01 ± 0.00	0.02 ± 0.00	1470	RI,MS
24	Germacrene	28.77	23986-74-5	C_15_H_24_	0.43 ± 0.02	0.46 ± 0.03	1733	
25	5-Oxatricyclo[8.2.0.04,6] dodecane, 4,12,12-trimethyl-9-methylene-, (1R,4R,6R,10S)-	34.86	1139-30-6	C_15_H_24_O	0.06 ± 0.00	0.14 ± 0.00	2001	RI^c^, MS
VIII. Cyclic terpene					0.5	0.66		
26	2,6-Octadiene, 2,6-dimethyl-	27.03	2792-39-4	C_10_H_16_	0.03 ± 0.00	0.04 ± 0.01	1659	RI,MS
27	Neryl acetate	28.51	141-12-8	C_12_H_20_O_2_	0.21 ± 0.01	0.19 ± 0.00	1722	RI,MS
28	Methyl hexadecanoate	43.77	112-39-0	C_17_H_34_O_2_	0.26 ± 0.01	0.30 ± 0.00	2210	RI^c^, MS
IX. Open-chain monoterpene					0.5	0.53		
Total (%)				100	100		
Yield (mg/g)				85.67 ± 0.73	114.02 ± 2.79	

*Essential oils extracted by UAPE and HD were determined in triplicate, and values are expressed as means ± SD. Values with the same letter are not significantly different p < 0.05 according to ANOVA*.

a *Compounds listed in order of elution from DB-WAX MS capillary column*.

b *Retention indices relative to C8-C20 n-alkanes on DB-WAX capillary column*.

c *Data identification on DB-WAX capillary column with literature data*.

d *ND, not detected*.

A higher yield of EO and short time-consuming was obtained by the UAPE technique. A reasonable principle behind such a phenomenon is likely owing to the UAPE being involved in the principle of acoustic cavitation, which promotes the destruction of plant cell walls, favoring solvent penetration, mass transfer, and the release of bioactive compounds quickly from its cells to the collector (32). Concerning traditional HD, the heat kinetic energy diffuses from solvent to the plant materials, resulting in the effective mass transfer occurring from the inner of plant materials to the outside (33). The proposed UAPE displays a significantly higher EO yield than HD, probably owing to the destruction of cellular structures of plant materials (34). However, different EO yields have been reported in previous studies (15). These discrepancies might be due to the differences in geographic location, cultivation, and sample preparation (35). In summary, compared with UAPE and traditional HD, the proposed UAPE was effective at increasing the plant EO yield.

### Characterization of the TCEO

**Figure 7:**
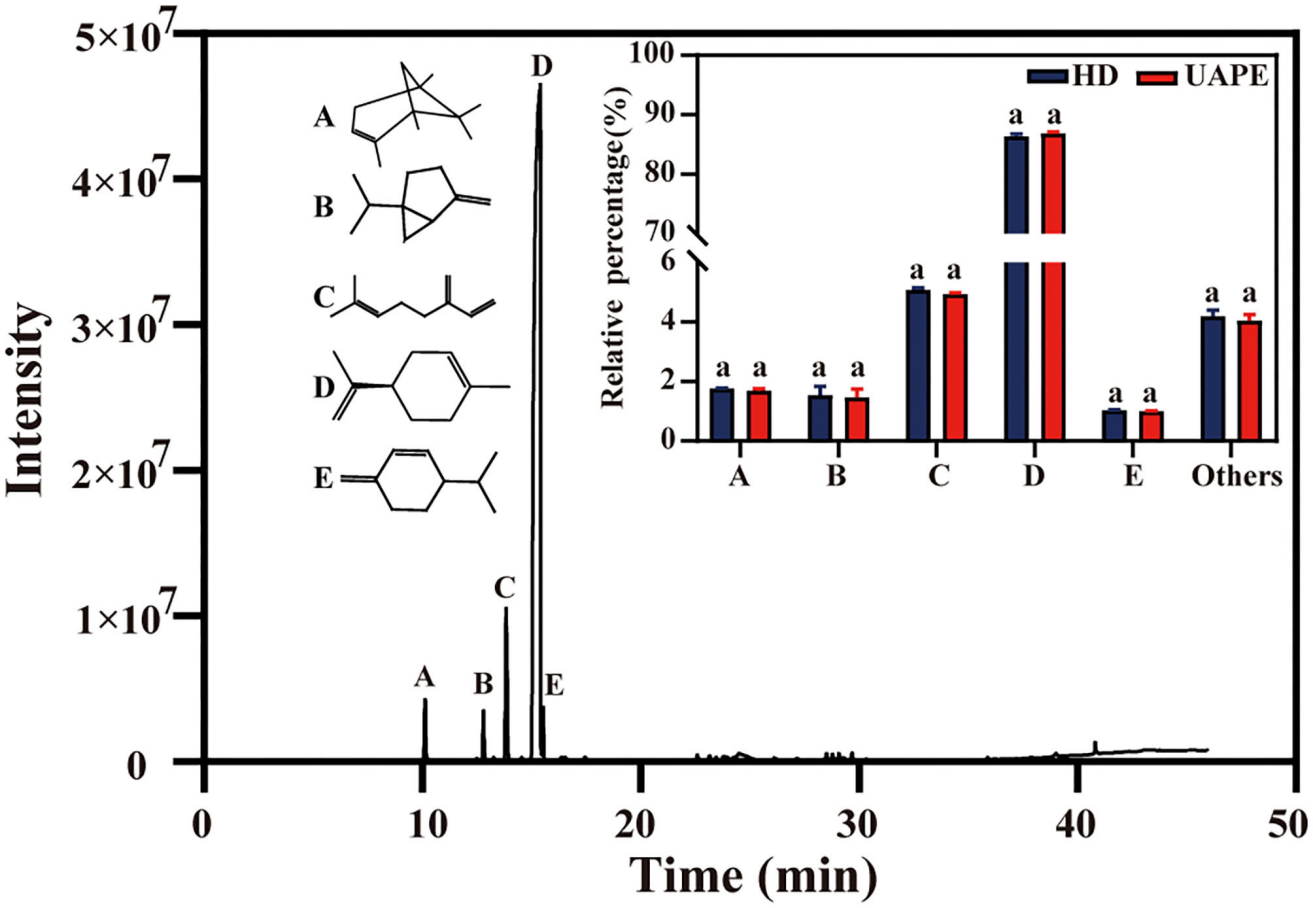
Total ion chromatogram of HD and UAPE. Inset: chemical structural formulas of five main compounds (α-pinene, sabinene, β-myrcene, d-limonene, β-phellandrene) and their relative percentage in the essential oils extracted by two methods. For each compound, values with the same latter are not significantly at the *p* < 0.05 level according to ANOVA.

The constituents of the EOs isolated from TC peels by UAPE and HD are presented in Figure 7 and Table 5. The GC-MS analysis results indicated that TCEO comprises various hydrocarbon-based components and metabolites linked with carbonyl and hydroxyl groups. In total, 28 compounds were identified and listed according to their Kovat's index, MS, and relative peak area as given in Table 5. Compared to the EO separated from UAPE and HD, no more obvious differences were found in the components of EOs obtained using the two methods. The presence of these constituents may be due to the long operation time for HD, which results in the materials being fully exposed to water and hence being subjected to oxidization, hydrolysis, and even other reactions. Similar phenomena were observed in the extraction of *Cinnamomum* camphor EO using a solvent-free microwave-assisted method (36). The significantly higher d-limonene content determined in the EO extracted by HD and UAPE was the most abundant compound in the EO of TC peels with the similarity relative amounts of 86.38 ± 0.31% and 86.75 ± 0.23%, respectively. Notably, the EO extracted using HD and UAPE possessed no significant difference in the high percentage of terpene (97.77% for HD and 97.78% for UAPE).

The type and percentage of terpene detected in the EO for HD and UAPE were cyclic monoterpenes (90.99, 91.16%), acyclic monoterpenes (5.09, 4.94%), acyclic sesquiterpenes (0.45, 0.47%), aldehydes (1.17, 1.08%), alcohols (0.59, 0.56%), dicyclic monoterpenes (0.64, 0.62%), sesquiterpenes (0.07, 0.02%), cyclic terpene (0.5, 0.66%), and open-chain monoterpene (0.5, 0.53%), respectively. A–E represent the chemical materials α-pinene, sabinene, γ-myrcene, d-limonene, and β-phellandrene. E represents the rest of the EO. All the above constituents display no significance (*p* < 0.05). Several studies regarding the citrus used to extract EO exhibited d-limonene as the predominant component, followed by other components like pinene, linalool, and farnesene (23, 26, 37–39).

### E-Nose

**Figure 8:**
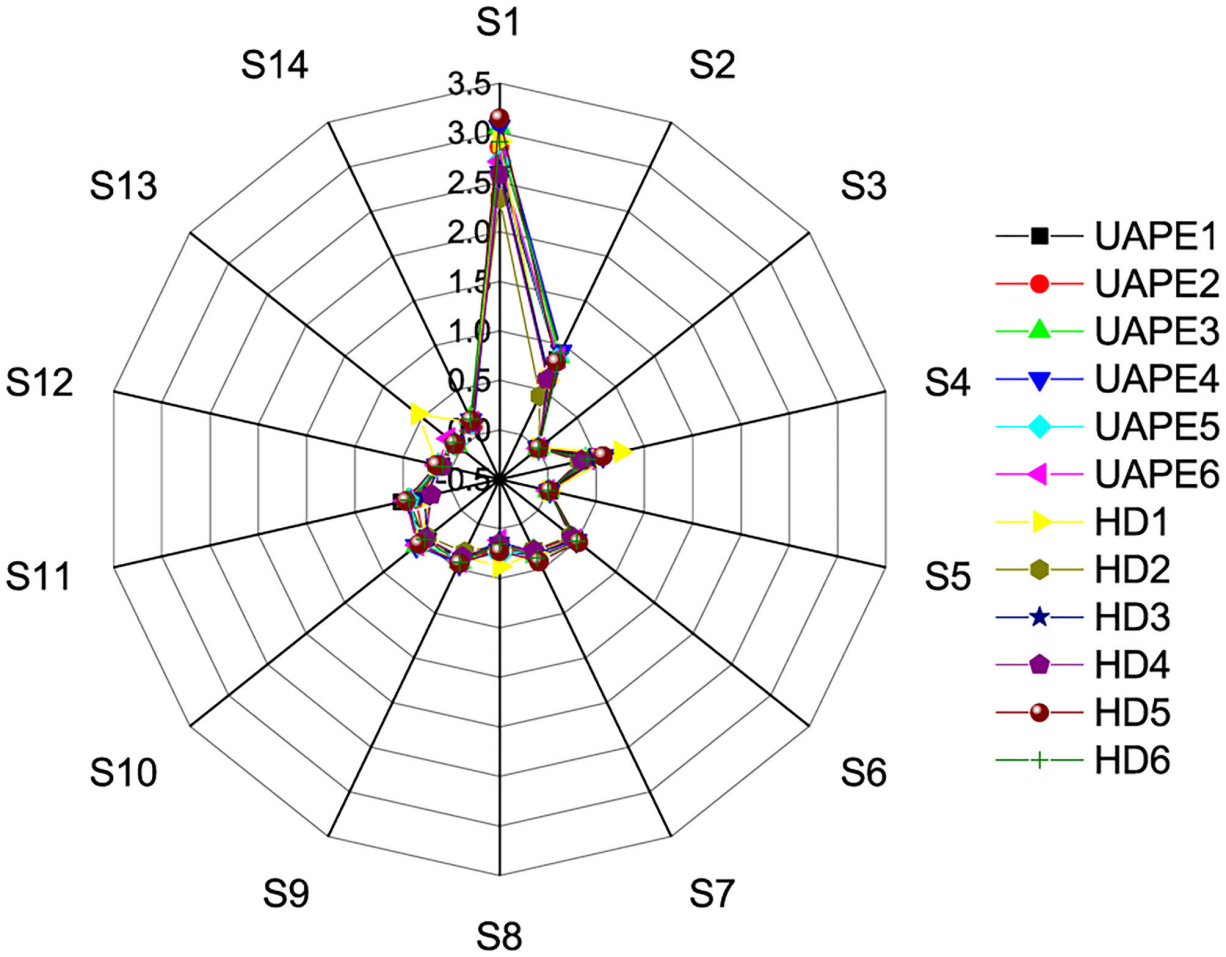
Response graphs of 14 sensors for flavor compound from TCEO by the UAPE and HD methods.

**Figure 9:**
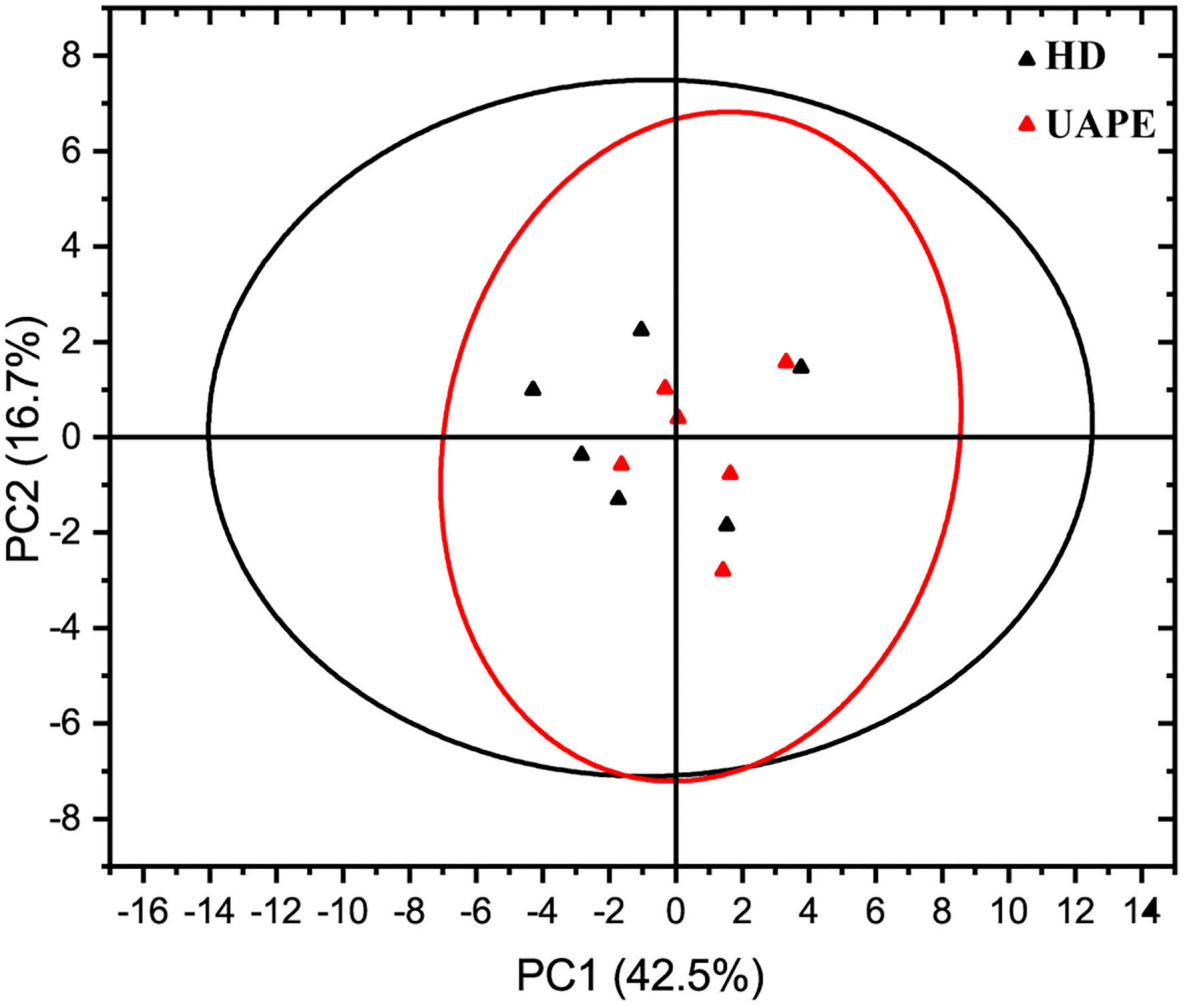
Principal component analysis of electronic nose data from TCEO by the UAPE and HD methods.

The principal component analysis (PCA) plot of the E-nose data is shown in **Figure 9**. The optimization of the 14 array sensors yielded data with a similar observation in the GC/MS. From the above results, the E-nose proposed in the research was capable of differentiating VOC of TCEO by differential extraction methods. Different extraction methods resulted in volatile aroma compound (VAC) differences. After 14 array sensor optimization, the chart figure displayed in Figure 8 shows that the sensors investigated obvious similarities between the 12 samples. The PCA result shows that the optimization sensors are generally correlated with 59.2% of the total variance containing the first principal component PC1 and the second principal component PC2, represented as PC1 42.5% and PC2 16.7%, respectively.

The PCA score plot shows a clear similarity to HD and UAPE. The VAC differences between the HD and UAPE from the TCEO were mainly reflected in the PC1 axis. Similarly, differences between the extraction methods by HD and UAPE from TCEO were mainly reflected in the PC2 axis. Furthermore, the difference between the 12 samples displayed in the chart graph (Figure 8) was similar. From the above result, we show that the UAPE treatment has less effect on the flavor of TCEO compared with the HD technique. Thus, ultrasound pretreatment was an important factor to be considered when designing EO extraction. Such a phenomenon was found in a previous study (29).

E-nose had potentially been used to preliminarily discriminate the VAC through sensor response analysis (40). Radar chart analysis and PCA are displayed in Figures 8, 9. From Figure 8, all the response sensors can be described as sensitive E-nose to detect VAC, wherein the extraction methods by the proposed UAPE and HD showed no significance. The principal component of the analysis chart is displayed in Figure 9. The TCEO extraction methods of UAPE and HD were densely distributed together and overlapped, illustrating no significant differences, and showed some similarities to the result of GC-MS (Table 5).

## Discussion

In this study, a comparison of EO extraction by HD and UAPE was proposed. Factors like the concentration of solid-liquid ratio, sodium chloride, extraction time, and ultrasound time were investigated. For the HD, the interaction between two factors was significant (*p* < 0.01). All the three-square terms showed significant impacts on the TCEO yield (*p* < 0.01). Whereas extraction time did not show any statistical significance. The same result was obtained by the UAPE method, which agreed with results obtained in other studies that showed no significant improvement on the yield of EO (*p* < 0.05) (41). The optimal conditions for the HD process were 20.32:1 solvent-solid ratio, 125.38 min extraction time, and sodium chloride concentration 1.15%, yielding a TCEO of 85.69 mg/g. Based on the predicted conditions, the verification experiment was carried out, and the TCEO yield was 85.67 mg/g, which was not significant to the predicted value (*p* < 0.05). While the optimal conditions for the UAPE process were 20.09:1 solvent-solid ratio, 20.7 min ultrasound-assisted, extraction 60.04 min, and sodium chloride concentration 1.62%, yielding a TCEO of 115.072 mg/g. Based on the predicted conditions, the verification experiment was carried out, and the TCEO yield was 114.02 mg/g. This was in agreement with the results obtained in other studies; therefore, the greater efficiency of UAPE over HD in terms of both increased EO yields and considerably shortened extraction times (26, 42).

The GC-MS analysis was employed to characterize the composition of TCEO extracted by the two methods; a total of 28 components were extracted from the TC peels, and the results demonstrated that a higher percentage of terpene compounds was determined in the EO. d-Limonene was the most vital component in the TCEO for 86.38% HD and 86.75% UAPE, respectively, followed by α-pinene, sabinene, β-myrcene, and β-phellandrene for 1.77, 1.55, 5.09, and 1.04% HD and 1.69, 1.47, 4.94, and 1.01% UAPE. A comparison with *Citrus sinensis* from central-eastern Sicily shows the main component is d-limonene (73.9–97%). Linalool, geraniol, and nerol were also found (43). EO displayed two types, both a volatile (93–96% of total) and a nonvolatile (4–7% of total) fraction. They include monoterpene limonene in the range of 25–53% in addition to the high quantities of oxygenated compounds, such as linalool (2–20%), linalyl acetate (15–40%), terpinene, and pinene (44).

Discrimination of EO extracted by HD and UAPE was investigated with 14 array sensor E-nose. Meanwhile, unsupervised machine learning PCA was used to discriminate the differences of extraction methods, and the first two principal components (PC1 42.5% and PC2 16.7%) were found and accounted for approximately 59.2% of total variances. The TCEO extraction methods by UAPE and HD were densely distributed together and overlapped, illustrating no significant differences, and showing some similarities to the result of GC-MS. Overall, UAPE performed better than HD concerning the EO yield, extraction time, and the extraction components showing no differences. Zhou Qi et al. multi-applied GC-MS, PCA, and E-nose to identify the discrepancy of extraction methods in rapeseeds. The PCA score plot shows a clear difference between the samples by PC1 and PC2, short UAPE extraction time, and no significant discrimination (45).

## Conclusion

A comparison extraction method by HD and UAPE was proposed, and the yield of EO increased significantly by 33.09% (*p* < 0.01) and time-consuming shortened 40 min. A total of 28 components were extracted from TC peels affirmed by GC-MS. d-Limonene was the vital compound followed by α-pinene, sabinene, γ-myrcene, and β-phellandrene. E-nose was employed to discriminate the differences through PCA and the radar chart. PCA and radar chart were densely distributed together and overlapped, illustrating no significant discrepancy, and were similar to the result of GC-MS. Overall, the UAPE method is a promising alternative to the traditional approach for TCEO extraction and has great application prospects for the isolation of EO from the plant matrix.

## Data Availability Statement

The original contributions presented in the study are included in the article/supplementary material, further inquiries can be directed to the corresponding authors.

## Author Contributions

GL: methodology, investigation, software, formal analysis, data curation visualization, writing-original draft, and editing. SL and JH: writing-review and editing. QZ: investigation and software. CQ, XM, and XG: data curation visualization. YL: formal analysis. TZ: supervision. PL: conceptualization, methodology, supervision, and validation. QG: conceptualization, methodology, supervision, validation, writing-review and editing, and funding acquisition. All authors have read and agreed to the published version of the manuscript.

## Funding

This study was funded by the National Key Research and Development Program of China (2017YFE0122300) and the Chinese Academy of Engineering Academy-Locality Cooperation Project (No. 2019-ZJ-JS-02).

## Conflict of Interest

The authors declare that the research was conducted in the absence of any commercial or financial relationships that could be construed as a potential conflict of interest.

## Publisher's Note

All claims expressed in this article are solely those of the authors and do not necessarily represent those of their affiliated organizations, or those of the publisher, the editors and the reviewers. Any product that may be evaluated in this article, or claim that may be made by its manufacturer, is not guaranteed or endorsed by the publisher.
